# Thermodynamic Activation Parameters for Chemical Reactions in Enzymes and Solution from Computer Simulations at a Single Temperature

**DOI:** 10.1021/acs.jctc.6c00368

**Published:** 2026-04-17

**Authors:** Florian van der Ent, Andrey O. Demkiv, Johan Åqvist

**Affiliations:** Department of Cell & Molecular Biology, 8097 Uppsala University, Biomedical Center, SE-751 24 Uppsala, Sweden

## Abstract

There is considerable interest in being able to address the temperature dependence of enzyme reactions by computer simulations. One reason for this is that enzymes that are adapted to different temperature regimes generally show distinct signatures in terms of their activation enthalpies and entropies, to the extent that it is basically possible to predict whether an enzyme is psychrophilic or mesophilic just by examining these activation parameters. The standard approach to this problem is to calculate reaction free energy profiles at a series of different temperatures. Computational Arrhenius plots can then be constructed from the data, analogous to the experimental procedure. This method has been shown to work well in a number of cases that have examined orthologous pairs of psychrophilic and mesophilic enzymes. The drawback is that the simulations have to be repeated at several temperatures. However, while multitemperature simulations may be computationally demanding they can be informative in revealing deviations from linear Arrhenius behavior. Another issue is that the calculated activation enthalpies obtained in this way cannot be readily decomposed into different energy terms. An alternative approach to the problem would be to just carry out free energy simulations at a single temperature and instead obtain the enthalpy profile by plain averaging of the total energy. The entropy term would then simply be calculated as the difference between free energy and enthalpy. This averaging approach was earlier considered unreliable due to convergence problems for the total energy, even for moderately sized systems. Here, we re-examine the performance of the averaging method for two solution reactions and one enzyme reaction and conclude that it works surprisingly well with sufficient data. This opens up new ways of analyzing nonlinearity of Arrhenius plots in terms of energetics, since the enthalpy is decomposable.

## Graphical abstract

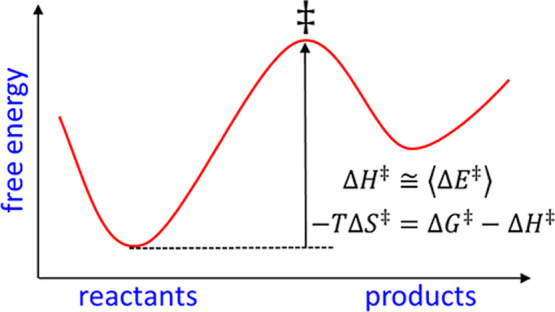

## Introduction

Calculations of thermodynamic activation parameters for chemical reactions in solution and enzymes from computational Arrhenius plots have become an important tool for exploring the energetics of enzyme catalysis. This approach relies on molecular dynamics (MD) free energy perturbation (FEP) simulations, whereby reaction free energy profiles are calculated at a series of different temperatures. Activation free energies are then plotted against temperature (or as Δ*G* ^⧧^/*T* vs 1/*T*) to obtain values of Δ*H* ^⧧^ and Δ*S* ^⧧^, similarly to what is done experimentally by plotting ln *k* vs 1/*T*.^1,2^ In most such applications, the reaction potential energy surface is represented by the empirical valence bond (EVB) model,^3,4^ which allows for the extensive sampling of configurations that is necessary to obtain convergent values of the thermodynamic activation parameters.^5^ The usefulness of this methodology is illustrated by the fact that the energetic origin of catalytic effects in enzymes, compared to their corresponding uncatalyzed solution reactions, can readily be dissected in terms of enthalpy and entropy.^6–9^ This is of particular importance in cases where the basis of the catalytic effect is under debate and may be difficult to explore by experiment.

Another area where the balance between activation enthalpy and entropy plays a major role regards the thermal adaptation of enzymes. Here, cold-adapted enzymes from psychrophilic or psychrotolerant species generally show distinctly different enthalpy–entropy signatures compared to orthologous mesophilic or thermophilic counterparts. The generally observed effect is that Δ*H* ^⧧^ has been reduced by evolution in cold-adapted enzymes at the expense of a more negative value of Δ*S* ^⧧^.^10–12^ As a consequence of this adaptation, the catalytic rate decay becomes less pronounced as the temperature is lowered. Moreover, in several cases computer simulations have shown that the origin of their shifted enthalpy–entropy balance has to do with a “softer” (more flexible) protein surface.^12,13^ There is thus a significant interest in reliable computational predictions of activation enthalpies and entropies and this has also recently allowed for redesigning the temperature dependence of cold-adapted enzymes in a rational way.^14,15^

The primary motivation for starting to explore the computational Arrhenius plot approach for enzymes in 2008,^16^ was that at that time calculation of Δ*H* ^⧧^ from averages of the total potential energy ⟨*U*_tot_^⧧^⟩ – ⟨*U*_tot_^R^⟩ at the transition and reactant states was deemed not to converge. For example, the averaging approach was tried for the peptidyl transfer reaction on the ribosome and, although qualitatively reasonable, it was found to give very large error bars.^17^ A subsequent analysis of ion hydration in 30 Å diameter water droplet also indicated that calculation of free energies at different temperatures was more reliable than collecting total energy averages at a single temperature.^18^ However, if Δ*H* ^⧧^ could be accurately obtained from simple averaging it would have the advantage that it could be directly decomposed into different contributions, e.g., Δ*U*_*rr*_^⧧^, Δ*U*_*rs*_^⧧^, Δ*U*_*ss*_^⧧^, where *r* and *s* denote the reacting groups (QM region in QM/MM language) and their surroundings, respectively. This is because the potential energies of most force fields are additive and Δ*U*_*ss*_^⧧^ could, of course, be further decomposed into protein and solvent contributions. It should also be noted that such a decomposition is not possible for either Δ*G* ^⧧^ or Δ*S* ^⧧^ due to the correlations involved in these quantities.^19^

In view of the development of computational power in that last 20 years, we decided herein to revisit the potential energy averaging approach for calculating activation enthalpies in order to reassess its performance, the idea being that it could perhaps suffice with MD/EVB/FEP calculations of Δ*G* ^⧧^ at a single temperature together with averaging of *U*_tot_, to obtain both Δ*H* ^⧧^ and Δ*S* ^⧧^. To this end, we examine here both two simple reactions in water and an ester hydrolysis reaction catalyzed by a small bacterial lipase, LipA from *Bacillus subtilis*.^15^ The results show that this approach works surprisingly well in our three test cases and that the calculated activation enthalpies and entropies compare well with those obtained from computational Arrhenius plots.

## Theoretical Background

With a two-state EVB model the system is represented by a 2 × 2 Hamiltonian matrix

1 $$\left[\begin{array}{ll} H_{11} & H_{12}\\ H_{21} & H_{22} \end{array}\right]\left(\begin{array}{l} c_{1}\\ c_{2} \end{array}\right)=E_{g}\left(\begin{array}{l} c_{1}\\ c_{2} \end{array}\right)$$

where *c* is the eigenvector and the ground-state energy (*E*_g_) is obtained as the lowest eigenvalue of the secular equation^3–5^

2 $$E_{g}=\frac{1}{2}\left(H_{11}+H_{22}\right)-\frac{1}{2}\sqrt{{\left(H_{11}-H_{22}\right)}^{2}+4H_{12}^{2}}$$

Here, the diagonal matrix elements *H*_11_ and *H*_22_ are classical force fields representing the reactant and product states of the reaction step. The off-diagonal element *H*_12_ = *H*_21_ represents the coupling or mixing of the two states and is often taken as a constant term, although it can also be given a distance or energy-gap dependent form (see below).^3,20,21^ *H*_11_ and *H*_22_ are standard force fields of the type

3 $$H_{ii}=U_{i}\left(R\right)=U_{rr}+U_{rs}+U_{ss}$$

where the subscripts *r* and *s* denote the reacting fragments, whose bonding and charge distribution changes during the reaction, and the surrounding environment, respectively. Bonds that form and break are represented by Morse potentials and a constant energy term, Δα, is added to *U*_2_ to account for the absolute energy difference between the two VB states.^3–5^ It should be noted here that the term *U*_*ss*_ (interactions within the surroundings) is equal for *U*_1_ and *U*_2_ and thus, in principle, fully enters into eq 2. While it cancels in free energy perturbation expressions (see below), it remains present in the absolute ground-state energy *E*_g_ and therefore affects enthalpy averages. It is also by far the largest energy term since it involves all interactions within and between the solvent and protein (when dealing with enzymes). For example, in the case of our lipase in a 62 Å diameter sphere of water *U*_*ss*_ is on the order of −40000 kcal/mol, while *U*_*rr*_ + *U*_*rs*_ is only about −600 kcal/mol.

In MD/EVB/FEP calculations of the free energy profile on the ground-state potential, a linear combination of *U*_1_ and *U*_2_ is used as an “umbrella”, or mapping, potential for obtaining the free energy on *E*_*g*_. That is, we use *U*_map_ (λ_*n*_) = (1 – λ_*n*_)*U*_1_ + λ_*n*_ *U*_2_ as a mapping potential with a number of discrete windows with different values of the mapping parameter λ_*n*_ ∈ [0,1]. The advantage with the FEP formula

4 $$\Delta G\left(\lambda_{m}\right)=-\beta^{-1}\sum_{n=0}^{m-1}\ln{\left\langle e^{-\beta \left(U_{map}\left(\lambda_{n+1}\right)-U_{map}\left(\lambda_{n}\right)\right)}\right\rangle }_{n}$$

is that *U*_*ss*_ vanishes since the exponent only contains energy differences between adjacent windows (β^–1^ = *RT*). The same goes for the umbrella sampling expression which connects Δ*G*(λ_*m*_) to the ground-state free energy profile Δ*G*_g_(*X*_*k*_), where the latter is expressed as a function of the discretized energy gap reaction coordinate *X*_*k*_ = Δ*U* = *U*_1_ – *U*_2_.^4^

5 $$\Delta G_{g}\left(X_{k}\right)=\sum_{m}p_{m}\left\{\Delta G\left(\lambda_{m}\right)-\beta^{-1}\ln{\left\langle e^{-\beta \left(E_{g}\left(\lambda_{m},X_{k}\right)-U_{map}\left(\lambda_{m},X_{k}\right)\right)}\right\rangle }_{m,k}\right\}$$

Here, the sum runs over those windows that contribute to the particular bin *X*_*k*_ and *p*_*m*_ is the normalized statistical weight of the *m*th sampling window in that bin.^1,5^ Again, we see that since both *E*_g_ and *U*_map_ contain the same *U*_*ss*_ term it will vanish from the free energy calculation and both Δ*G*(λ_*m*_) and Δ*G*_g_(*X*_*k*_) are thus relative free energy profiles.

However, from eq 2 we see that *E*_g_ itself, of course, contains the full *U*_*ss*_ term that needs to be converged in order for the corresponding calculation of Δ*H*_g_(*X*_*k*_) ≅ ⟨Δ*E*_g_ (*X*_*k*_)⟩ to be meaningful. And here there is no perturbation approach to help us. We will thus try to directly evaluate Δ*H*_g_(*X*_*k*_) from MD/EVB/FEP simulations at a single temperature simply by again discretizing and binning the total ground-state energy *E*_g_(*X*_*k*_), in the same way as done for Δ*G*_g_(*X*_*k*_), from the data collected during multiple replicate FEP simulations. The activation entropy contribution can then just be obtained from the free energy simulations as −*T*Δ*S* ^⧧^ = Δ*G* ^⧧^ – Δ*H* ^⧧^.

## Results

### Simulations of a Unimolecular Reaction in Solution

As a first test case we will consider the unimolecular Claisen rearrangement of chorismate to prephenate in water (Figure 1), which is the same reaction as that catalyzed by the chorismate mutase enzyme.^22,23^

**Figure 1:**
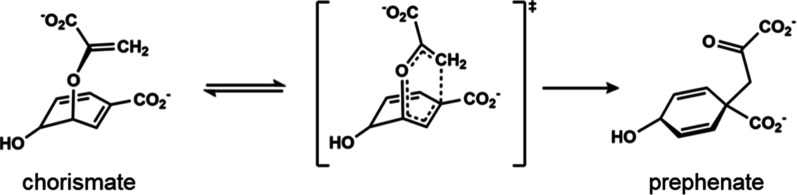
Intramolecular rearrangement of chorismate to prephenate, where the pseudodiaxial conformation of chorismate is indicated.

The reaction is represented by a two-state EVB model and parametrized against DFT calculations as done before,^23^ but here we employ a Gaussian energy-gap dependent form of the off-diagonal element, *H*_12_ = *A* × *exp*(–*b*Δ*U* ^2^), instead of a constant *H*_12_. One reason for this is that chorismate is known to be in equilibrium between an extended pseudodiequatorial and a more compact transition state-like pseudodiaxial conformation (shown in Figure 1), reached by rotation around the breaking C–O bond.^22,24^ The pseudodiequatorial conformation is energetically favored and has a significantly larger distance between the two bond-forming carbon atoms. It thus seems more appropriate that the coupling between the two EVB states should be weaker in that case. By starting 60 multiple MD/EVB/FEP simulations from the approximate transition state (λ = 0.5) at each of five different temperatures (293–313 K), we filtered out those (15–30%) at each temperature that did not sample the entire reaction coordinate and got ‘trapped’ in the pseudodiaxial conformation (Figure 2a). A typical free energy profile from the simulations is shown in Figure 2b and the average values of Δ*G* ^⧧^ and Δ*G* ^0^ for the reaction from all simulations are 24.5 and −13.2 kcal/mol, respectively at 25 °C.^22,23^ From the accumulated data at different temperatures we calculated an Arrhenius plot (Figure 2c), which gives estimates of Δ*H* ^⧧^ = 21.3 and *T*Δ*S* ^⧧^ = −3.2 kcal/mol, similar to the results obtained earlier^23^ and to the experimental values of Δ*H* ^⧧^ = 20.7 and *T*Δ*S* ^⧧^ = −3.8 kcal/mol.^22^

**Figure 2:**
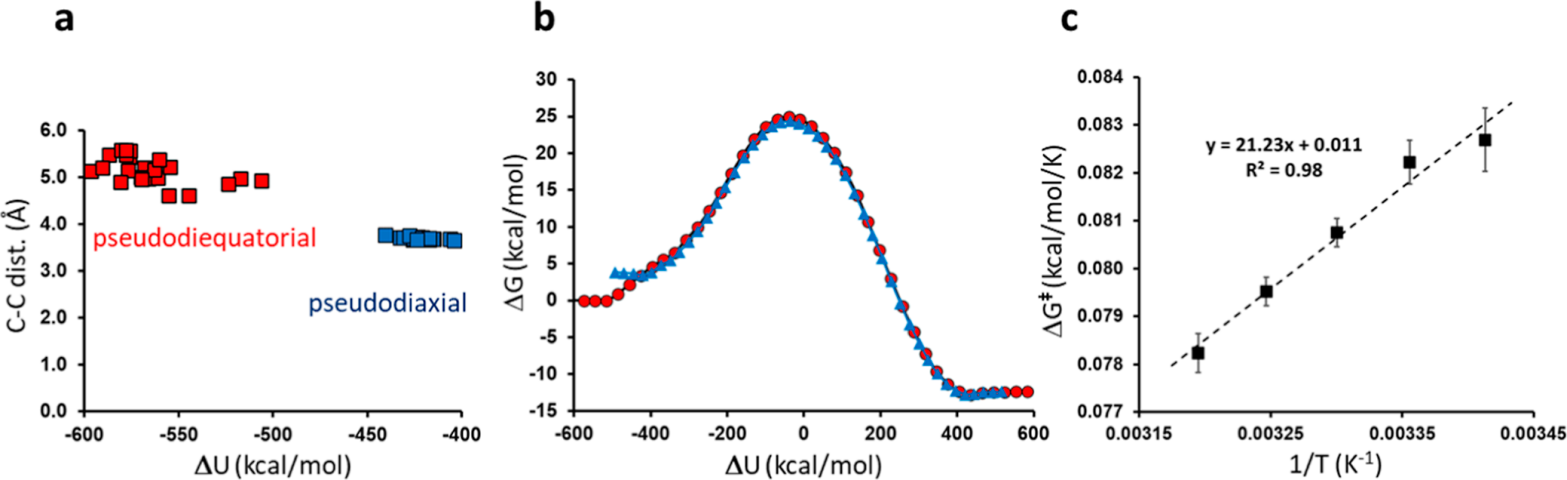
(a) Plot of the bond-forming C–C distance at the reactant minimum from 60 independent free energy simulation, as a function of the energy gap reaction coordinate, where the pseudodiequatorial conformation (red) is to the left and the higher energy pseudodiaxial conformation corresponds to the isolated cluster to the right (blue). (b) A representative free energy profile calculated for the chorismate reaction from the pseudodiequatorial minimum (red) and a corresponding profile from the trapped pseudodiaxial conformation (blue). (c) Computed Arrhenius plot for the reaction from the pseudodiequatorial conformation at five temperatures (error bars ±1 s.e.m.). The average s.e.m. and 95% confidence interval for the calculated free energy barriers are ±0.13 and ±0.25 kcal/mol, respectively.

We then calculated the enthalpy profile for the reaction directly from ⟨*E*_g_(*X*_*k*_)⟩ for the 60 replicate simulations at 298 K and the result is shown in Figure 3a (corresponding to a total of about 6.3 million energy points). The activation enthalpy obtained from this analysis is Δ*H* ^⧧^ = 21.2 ± 0.3 kcal/mol and should be measured from the value of the reaction coordinate corresponding to the minimum of Δ*G*_g_(*X*_*k*_) to its maximum. This gives an estimated value of *T*Δ*S* ^⧧^ = −3.3 kcal/mol by using the average value of Δ*G* ^⧧^ at 298 K. The direct approach of calculating Δ*H* ^⧧^ at a single temperature thus gives very reasonable results compared to the Arrhenius plot. It can be seen from Figure 3a that there indeed is a slight minimum in the Δ*H*_g_ profile also for the pseudodiaxial conformation before the transition state is reached. The enthalpy profile can further be decomposed into its *U*_*rr*_ + *U*_*rs*_ and *U*_*ss*_ contributions (Figure 3b,c). The decomposition shows the general trend that the exothermicity of the reaction derives from the interactions involving the reacting groups (Figure 3b). Here, one can examine the raw energies and see that it is mainly the electrostatic part of *U*_*rs*_ that is responsible for the effect. This energy term is considerably more favorable for prephenate than chorismate, but is partly compensated by an unfavorable change in the *U*_*rr*_ term. This is due to the more localized charge distribution in prephenate and also a generally slightly smaller distance between the two carboxylate groups. The trend is, however, opposed by more unfavorable solvent–solvent interactions (Figure 3c). It is also evident that the two conformational minima in water are intrinsic to the molecule (Figure 3b), but correspond to local maxima in the *U*_*ss*_ interactions (Figure 3c), which illustrates the compensatory nature of solute and solvent interactions. It should, however, be noted here that as far as *E*_g_ is concerned, *U*_*rr*_ + *U*_*rs*_ is not strictly separable into components due the energy gap dependence on this quantity and its appearance in the square root of eq 2. The raw *U*_*rr*_ and *U*_*rs*_ energies are nevertheless informative as discussed above.

**Figure 3:**
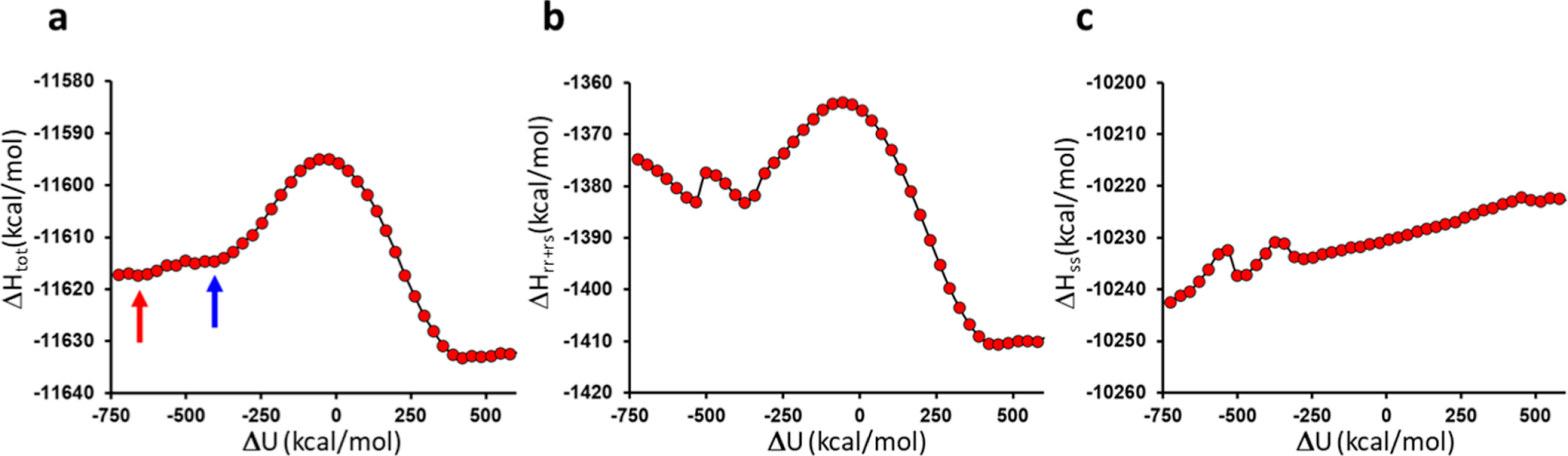
(a) Calculated enthalpy profile for the reaction from 6.3 million energy points at 298 K (s.e.m. 0.1–0.3 kcal/mol), with the two reactant conformations indicated. (b) Contribution from the *U*_*rr*_ + *U*_*rs*_ term to the total enthalpy along the reaction coordinate and (c) the corresponding contribution from the solvent interactions, *U*_*ss*_.

### Simulations of the Solution Reference Reaction for Ketosteroid Isomerase

Our second test case is the acetate catalyzed proton abstraction from 5-androstene-3,17-dione in water (Figure 4a). This bimolecular reaction corresponds to that catalyzed by the enzyme ketosteroid isomerase, where an aspartate residue abstracts the 4β proton from the steroid substrate.^25,26^ The usual procedure for EVB simulations of a bimolecular reaction is to apply a distance restraint between the reacting atoms, in this case the proton donor (C) and acceptor (O) atoms to keep them within contact distance. The approximation made then is that the proton acceptor will have the same concentration as the surrounding solvent (55 M) and, hence, the experimental forward (*k*_1_) and backward (*k*_–1_) rate constants will be corrected to become 55× faster than when given in units of M^–1^ s^–1^ (see ref 5. for a detailed discussion).

**Figure 4:**
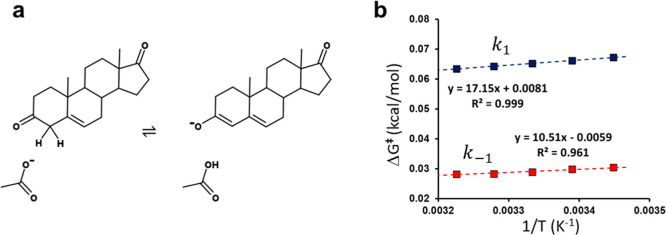
(a) The acetate catalyzed proton abstraction from 5-androstene-3,17-dione. (b) Calculated Arrhenius plots for the forward (blue) and reverse (red) reactions from 60 independent MD/EVB/FEP simulations at each temperature.

Our earlier simulations of this system showed that with this procedure one obtains activation enthalpies and entropies from computational Arrhenius plots in excellent agreement with the experimentally derived ones.^5^ Here, we followed our earlier protocol and again ran MD/EVB/FEP simulations of the reaction at five different temperatures (290–310 K) to evaluate the temperature dependence (Figure 4b). We additionally explored the effect of different trajectory lengths on both the Arrhenius plot and the ⟨*E*_g_(*X*_*s*_)⟩ calculations. Hence, 60 replicate simulations were run at each temperature, each with 1.05 ns, 5.25 and 10.5 ns of sampling per free energy profile. Our standard protocol with 60 × 1.05 ns per temperature yields values of Δ*H* ^⧧^ = 17.1 and *T*Δ*S* ^⧧^ = −2.4 kcal/mol for *k*_1_ and *H* ^⧧^ = 10.5 and *T*Δ*S* ^⧧^ = +1.8 kcal/mol for *k*_–1_ (Figure 4b). The corresponding experimental values are Δ*H* ^⧧^ = 16.4 and *T*Δ*S* ^⧧^ = −2.6 kcal/mol for the forward reaction and Δ*H* ^⧧^ = 9.6 and *T*Δ*S* ^⧧^ = +1.7 kcal/mol for the reverse one.^25,26^

A typical free energy profile from one of the simulations at 300 K for the reaction is shown in Figure 5a. The three different trajectory lengths yield a total of about 0.63, 3.15, and 6.3 million energy data points (Mpts), respectively, for the calculations of ⟨*E*_g_(*X*_*s*_)⟩. The reaction enthalpy profiles for these three cases are shown in Figure 5b and the results are summarized in Table 1. It can be seen that forward and reverse activation enthalpies are quite stable and in rather good agreement both with those derived from the computational Arrhenius plot and from experiments. Moreover, the experimental enthalpies are subject to relatively large error bars of 1.8–1.9 kcal/mol,^26^ wherefore all calculated values actually fall within this range of their values. For this relatively simply system, we do not see any distinct advantage with the longer simulations, apart from smaller error bars for the direct Δ*H* ^⧧^ calculations, and the Arrhenius plots do not really improve with longer simulations.

**Figure 5:**
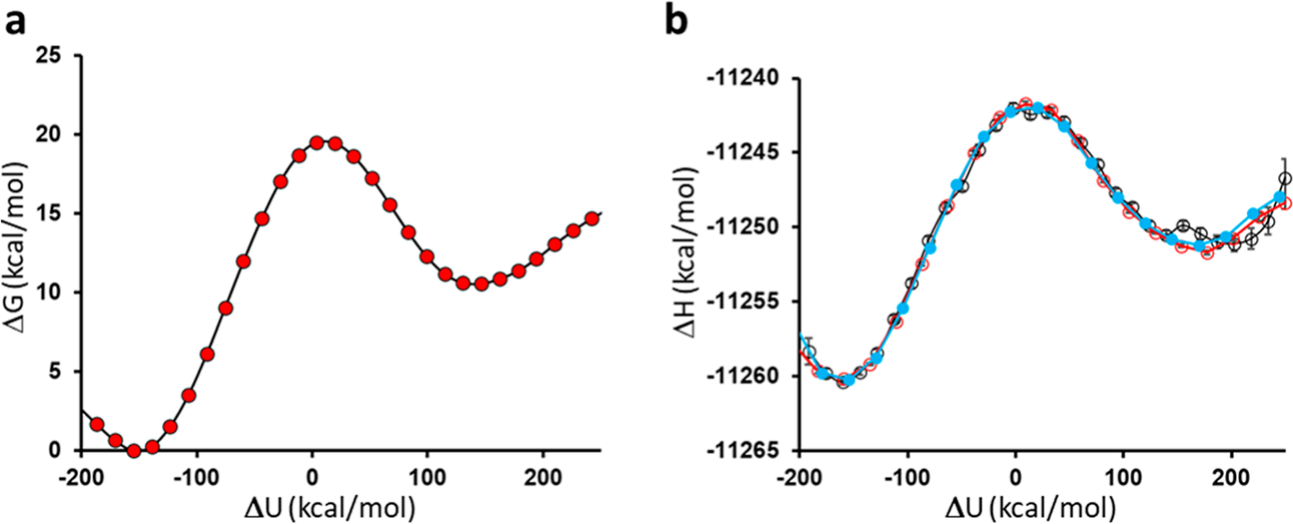
(a) Representative free energy profile for the acetate catalyzed isomerization reaction at 300 K. (b) Calculated enthalpy profiles for the reaction with 0.63 (black), 3.15 (red) and 6.3 (blue) million energy data points (error bars ±1 s.e.m.).

**Table 1:** Calculated and Experimental^25,26^ Activation Enthalpies and Entropies for the Acetate Catalyzed Proton Transfer Reaction in Water at 300 K (kcal/mol)^a^

	Arrhenius			direct calculation			
reaction	1.05 ns	5.25 ns	10.5 ns	0.63 Mpts	3.2 Mpts	6.3 Mpts	experiment
Δ*H* ^⧧^							
*k*_1_	17.1 ± 0.3	17.9 ± 0.7	18.3 ± 0.6	17.6 ± 0.4	18.2 ± 0.2	18.0 ± 0.1	16.4 ± 1.9
*k*_–1_	10.5 ± 1.2	9.5 ± 1.1	8.5 ± 1.5	8.1 ± 0.5	9.1 ± 0.2	8.6 ± 0.1	9.6 ± 1.8
*T*Δ*S* ^⧧^							
*k*_1_	–2.4 ± 1.8	–1.7 ± 0.7	–1.3 ± 0.6	–1.9 ± 0.4	–1.5 ± 0.2	–1.6 ± 0.1	–2.6 ± 1.8
*k*_–1_	1.8 ± 1.2	0.7 ± 1.1	–0.3 ± 1.5	–0.6 ± 0.5	0.3 ± 0.2	–0.1 ± 0.1	1.7 ± 0.3

a The experimental activation free energies and entropies have been corrected to represent a 55 M standard state, as described in the text. The Arrhenius plots are carried out with different trajectory lengths and the corresponding amount of data (Mpts = million energy points) is indicated for the direct calculations at 300 K. Error bars for Arrhenius values are the asymptotic standard errors from linear regression, otherwise ±1 s.e.m.

### Simulations of the Enzyme LipA from *B. subtilis*

As a final test case we wanted to examine the performance of direct activation enthalpy calculations for an enzyme reaction and here we consider the water attack on the acyl-enzyme intermediate in the small lipase LipA (183 amino acids). We have shown earlier, both with kinetic experiments and QM/MM calculations, that hydrolysis of the acyl-enzyme is rate-limiting with the *p*-nitrophenyl butyrate substrate.^27^ Nucleophilic attack of a water molecule on the butyrate ester formed with Ser77 of the catalytic triad (acyl-enzyme) results in a transient tetrahedral intermediate (Figure 6a). Breakdown of this intermediate then leads to release of butyric acid and restoration of the enzyme to its initial catalytic state. The calculated Arrhenius plot from our earlier MD/EVB/FEP simulation for this reaction step is shown in Figure 6b.^15^ It gives values of Δ*H* ^⧧^ = 9.8 and *T*Δ*S* ^⧧^ = −5.1 kcal/mol at 25 °C. The corresponding experimental values measured for hydrolysis of the acyl-enzyme are Δ*H* ^⧧^ = 9.1 and *T*Δ*S* ^⧧^ = −6.3 kcal/mol, but these are predicted by the EVB simulations to mainly reflect breakdown of the tetrahedral intermediate, for which the calculated activation free energy barrier is about 0.5 kcal/mol higher than the water attack.^15^ As shown earlier, the EVB simulations for this enzyme capture the experimental results extraordinarily well and we were able to design more efficient enzymes with altered temperature optima based on the calculations.^15^

**Figure 6:**
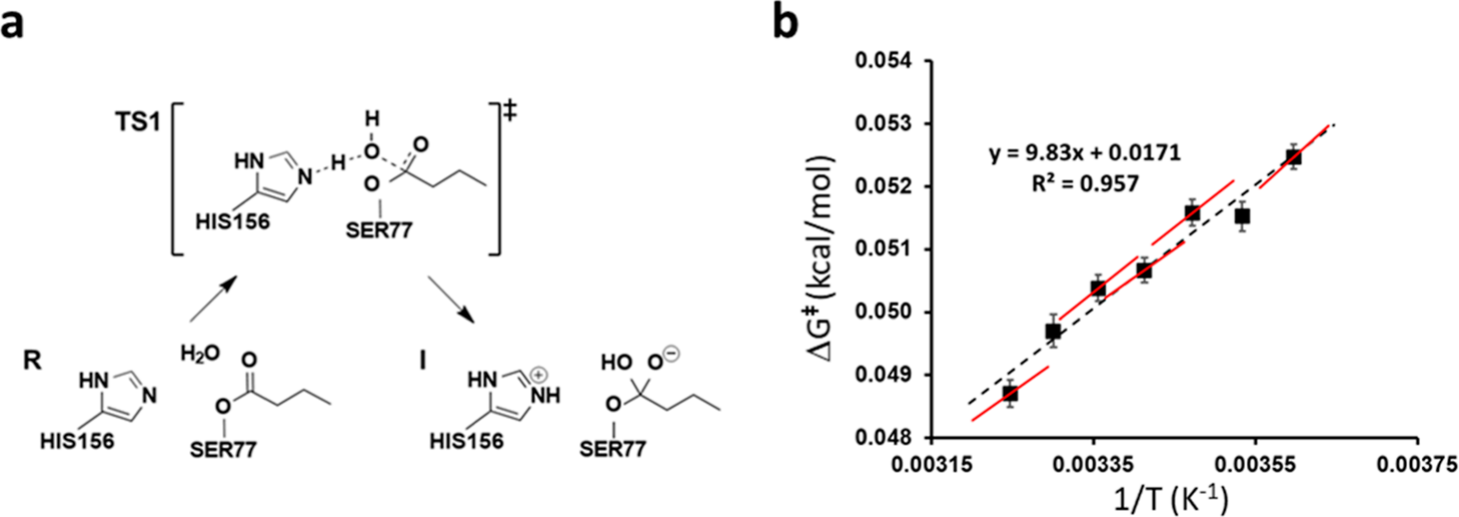
(a) The initial reaction step in hydrolysis of the acyl-enzyme in LipA which yields a transient tetrahedral intermediate. (b) Calculated Arrhenius plot for this reaction step from MD/EVB/FEP calculations at seven temperatures (error bars ±1 s.e.m.). The red lines are the local slopes obtained from direct Δ*H* ^⧧^ calculations (see below), while the dashed black line is the linear regression for all seven points.

Here, we use our earlier simulation data for the first step in hydrolysis of the acyl-enzyme for direct calculations of Δ*H* ^⧧^. The EVB/FEP simulations involved 50 replicate free energy profiles with a total of 155 ns of MD sampling at each of seven temperatures in the range 278–308 K.^15^ For any given temperature, this allowed us to collect about 3.04 million energy points that were used for the enthalpy calculations. From the data at 298 K we thus obtained values of Δ*H* ^⧧^ = 10.3 and *T*Δ*S* ^⧧^ = −4.7 kcal/mol, which compares well with those derived from the Arrhenius plot (Figure 6b). The two different ways of obtaining activation parameters thus agree in that about 2/3 of the free energy barrier derives from enthalpy and 1/3 from entropy. The calculated reaction enthalpy profile and its components are shown in Figure 7 together with the convergence of Δ*H* ^⧧^ as a function of the amount of data used in the calculation (Figure 7b). The compensatory nature of the *U*_*rr*_ + *U*_*rs*_ and *U*_*ss*_ contributions is again evident (Figure 7c,d). Here, *U*_*rr*_ + *U*_*rs*_ drops by about 8 kcal/mol between the reactant and product minima, while *U*_*ss*_ increases by about 11 kcal/mol, and the total enthalpy change is thus ∼3 kcal/mol. Hence, even though the absolute magnitudes of the two components are very different their change along the reaction is of similar magnitude, which may indicate that the compensation effect is rather local. In this case the origin of the favorable *U*_*rr*_ + *U*_*rs*_ interactions in the tetrahedral intermediate is the zwitterionic state that is formed, which in turn is associated with unfavorable interactions within the surrounding protein and solvent.

**Figure 7:**
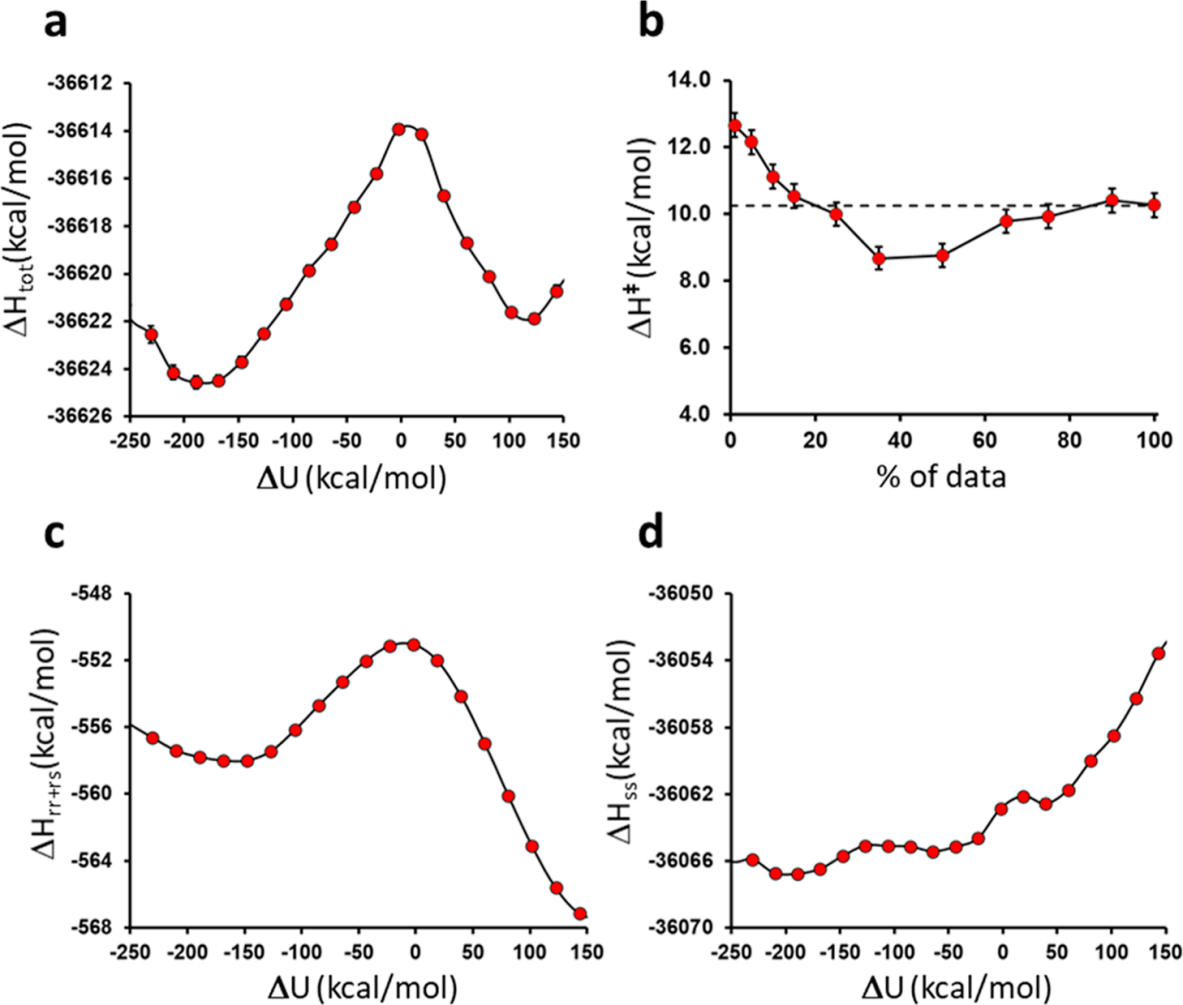
(a) Reaction enthalpy profile for the initial step of hydrolysis of the acyl-enzyme obtained from direct enthalpy calculations at 298 K (∼3 million energy data points). (b) Convergence of Δ*H* ^⧧^ as a function of the percentage of the amount of data used (error bars ±1 s.e.m.). (c,d) Decomposition of the total enthalpy profile into its (c) *U*_*rr*_ + *U*_*rs*_ and (d) *U*_*ss*_ terms.

Considering that the entire enzyme here is solvated in a 62 Å diameter water droplet, with total potential energy of ∼36000 kcal/mol, it is perhaps not so surprising that both the Arrhenius plot and the enthalpy profile are somewhat less accurate than in the two simpler solution reactions discussed above. Nevertheless, we find it rather remarkable that the direct calculation of Δ*G* ^⧧^, Δ*H* ^⧧^ and *T*Δ*S* ^⧧^ for an enzyme reaction at a single temperature gives such reasonable results. This is of particular interest in cases where the Arrhenius plot shows a distinct curvature since it may give a better understanding of why the activation enthalpy varies along the reaction.^15,28^ That is, by evaluating Δ*H* ^⧧^ at several temperatures where the Arrhenius plot has different slope, its energetic origin could be examined. As a first test of this idea we also calculated Δ*H* ^⧧^ and *T*Δ*S* ^⧧^ at a few other temperatures from our earlier simulations and these results are summarized in Table 2. The calculated Δ*H* ^⧧^ values between 288 and 308 K are all within 1 kcal/mol of that from the Arrhenius plot, while the value at 278 K appears to be a slight outlier. That is, although it lies perfectly on the regression line in the Arrhenius plot (rightmost point in Figure 6b), it deviates by more than the combined error from the Arrhenius values of 9.8 kcal/mol. However, the average value over the entire temperature range of Δ*H* ^⧧^ = 10.0 kcal/mol is in good agreement with the 9.8 kcal/mol obtained from the Arrhenius plot.

**Table 2:** Calculated Activation Enthalpies and Entropies for the First Step in Hydrolysis of the LipA Acyl-Enzyme (kcal/mol).^a^

reaction	278 K	288 K	293 K	298 K	308 K	Arrhenius
Δ*H* ^⧧^	11.7 ± 0.4	10.0 ± 0.3	9.0 ± 0.3	10.3 ± 0.4	9.2 ± 0.4	9.8 ± 0.9
*T*Δ*S* ^⧧^	–2.9 ± 0.4	–4.8 ± 0.3	–5.8 ± 0.3	–4.7 ± 0.4	–5.8 ± 0.4	–5.1 ± 1.0

a Error bars for the calculated enthalpies and entropies denote the standard error of the mean (s.e.m.) from 50 replicate simulations, while those for the Arrhenius parameters are the asymptotic standard errors from linear regression. The Arrhenius entropy term (*T*Δ*S* ^⧧^) is given at 298 K.

## Conclusions

Herein, we re-examined the idea of calculating activation enthalpies and entropies from simulations of reaction free energy profiles at a single temperature. The main obstacle here is that the total energy of the system needs to be properly converged in order for the calculated average ⟨*E*_g_(*X*_*s*_)⟩ along the reaction coordinate to be meaningful. If so, the entropy term −*T*Δ*S* ^⧧^ can then be simply obtained as the difference between free energy and enthalpy. As it turns out for our three test cases, the approach appears to work surprisingly well given that sufficient sampling is carried out when calculating the free energy profile. Here, it is paramount that a relatively large number of simulation replicas are used (50–60 in our case) in order to push down the error bars. This is likely more important than the length of the individual trajectories, provided that the system is well equilibrated.

It is of particular interest if this method works well for enzyme reactions, since it would then allow a more detailed analysis of cases where the Arrhenius plot deviates from linearity.^28^ For cases with curved Arrhenius plots, that often translate to a rate optimum occurring at lower temperatures than the measured *T*_m_, it would become possible to further examine the energetic origins of the effect as a complement to structural analysis of the simulations.

In principle, the direct calculation of Δ*H* from the total energy averages is not limited to EVB simulations, but could equally well be applied to any FEP or umbrella sampling calculation, where Δ*H*(λ) would be obtained in the same way (a criterion would, however, presumably be that the protein is fully solvated and not subjected to artificial restraints). This approach was, for example, demonstrated by us in the simple test case of ion hydration.^18^ In the more challenging case of free energy calculations for protein–ligand binding, a problem is that thermodynamic cycles need to be used, which would require high accuracy for the total energy in each leg of the cycle. Nevertheless, decomposition of relative or absolute binding free energies into their enthalpic and entropic contributions in such calculations is of considerable interest.

## Methods

All MD simulations were carried out as described earlier for the chorismate rearrangement in water,^23^ the reference reaction for ketosteroid isomerase^5^ and the hydrolysis of the acyl-enzyme intermediate in LipA,^15^ using the program Q (v. 5)^29^ and the standard OPLS-AA/M force field^30^ with parameters generated from Schrödinger’s ffld_server.^31^ The simulations used a 1 fs time step and the Berendsen thermostat with a temperature relaxation time of 10 fs.^32,33^ Briefly, the calculations were done as follows.

### Chorismate to Prephenate Rearrangement

The reaction was simulated in a 40 Å diameter droplet of TIP3P water.^30^ Following successive heating of the system to the target temperature and 500 ps of equilibration, MD/EVB/FEP calculations were initiated from the approximate transition state with λ = 5.0 and propagated toward the end-points with λ = 1.0 and λ = 0.0 in 21 discrete windows. Each free energy profile (eq 5) comprised 10.5 ns of sampling and 60 replicate such simulations of were carried out at each of five different temperatures in the range 293–313 K. The EVB parameters were fitted to the data at 298 K to reproduce the target values of Δ*G* ^⧧^ = 24.5 and Δ*G* ^0^ = −13.2 kcal/mol. This yielded values of the EVB parameters Δα = 24.4 and *H*_12_ = 74.0 × *exp*(–10^–5^Δ*U* ^2^), using a Gaussian form of *H*_12_. These parameters were then also used to calculate the enthalpy profile ⟨*E*_g_(*X*_*s*_)⟩ from the ∼6.3 M energy data points collected at 298 K.

### Acetate Catalyzed Deprotonation of 5-Andro-3,17-dione

The simulations were carried out exactly as described earlier^5^ with the reactants in a 40 Å diameter water sphere, again initiating the free energy calculations from λ = 0.5 with 21 evenly spaced sampling windows. The EVB parameters were fitted to the data collected at 300 K (Δα = −99.45 and a constant *H*_12_ = 52.6) in order to reproduce the target values of Δ*G* ^⧧^ = 19.6 and Δ*G* ^0^ = 10.9 kcal/mol for the forward reaction.^5^ At each of five temperatures (290–310 K) 60 replicate FEP calculations were carried out and here we also examined three different trajectory lengths of 1.05, 5.25, and 10.5 ns per individual free energy profile. Enthalpy profiles ⟨*E*_g_(*X*_*s*_)⟩ were calculated with each of the different trajectory lengths from the corresponding data at 300 K.

### Enthalpy Calculations for the LipA Reaction

Here, we used the earlier simulation data obtained in ref 15. for the mesophilic lipase LipA from *B. subtilis* (denoted mLipA in ref 15.). As the hydrolysis reaction of the acyl-enzyme involves two steps with slightly different free energy barriers, we herein only consider the initial nucleophilic attack by a water molecule on the butyrate ester formed with Ser77 of the catalytic triad. The mLipA model was based on the crystal structure 1R4Z.^34^ The tetrahedral intermediate was constructed based on our crystallographically determined structure with a covalent inhibitor of the 81% identical cold-adapted lipase from *Bacillus pumilus*.^27^ The MD/FEP/EVB simulations were in this case performed with enzyme solvated in a 62 Å diameter water droplet that fully covers the protein. Following energy minimization, heating and equilibration as, the FEP calculations were carried out with 31 discrete windows (100 ps per window) and 50 independent replicas per temperature (278, 288, 293, 298, and 308 K),^15^ which adds up to a total of 155 ns of sampling (∼3.04 million energy points) per temperature point. From this data we calculated ⟨*E*_g_(*X*_*s*_)⟩ at each of the five temperatures. The values of the EVB parameters were Δα = 253.1 and *H*_12_ = 54.3 kcal/mol.^15^
